# A case of HER-2-positive recurrent breast cancer showing a clinically complete response to trastuzumab-containing chemotherapy after primary treatment of triple-negative breast cancer

**DOI:** 10.1186/1477-7819-9-146

**Published:** 2011-11-07

**Authors:** Hideo Shigematsu, Takayuki Kadoya, Yoshie Kobayashi, Keiko Kajitani, Tatsunari Sasada, Akiko Emi, Norio Masumoto, Rumi Haruta, Tsuyoshi Kataoka, Miyo Oda, Kouji Arihiro, Morihito Okada

**Affiliations:** 1 Department of Breast Surgery, Hiroshima University Hospital, Hiroshima, Japan; 2 Department of Pathology, Hiroshima University Hospital, Hiroshima, Japan

**Keywords:** discordance, HER-2, trastuzumab, recurrent breast cancer

## Abstract

We report a case of HER-2-positive recurrent breast cancer showing a clinically complete response to trastuzumab-containing chemotherapy 6 years after primary treatment of triple-negative breast cancer. The primary tumor was negative for HER-2 as determined by immunohistochemistry (IHC) and fluorescence in situ hybridization (FISH) (1+, and ratio, 1.1), but examination of the recurrent lymph node metastasis showed positivity for HER-2 by FISH (ratio, 5.2). No lesions were detected in either her left breast or in other organs, and the patient was diagnosed as having HER-2-positive recurrent disease. Combination chemotherapy using weekly paclitaxel and trastuzumab was initiated, and a clinically complete response was achieved. This report suggests the benefit of routine evaluation of HER-2 status in recurrent breast cancer with the introduction of HER-2-targeting agents.

## Background

Recently, several reports have demonstrated that there are substantial discordances in hormone receptor expression and HER-2 status between primary tumors and metastases, which could alter the treatment and prognosis of recurrent breast cancer [1-5]. The discordance between HER-2 status in primary tumors and in metastatic sites occurs less frequently than the discordance between hormonal receptors [2-4,6], and the impact on prognosis is still unknown [7]. We here report on a case of recurrent HER-2-positive breast cancer showing a clinically complete response to trastuzumab-containing chemotherapy 6 years after primary treatment of triple-negative breast cancer.

## Case presentation

**Figure 1:**
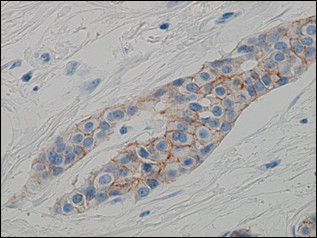
**Immunohistochemical staining for HER-2 protein overexpression using the HercepTest showed weak membrane staining in 30% of the primary tumor, corresponding to a 1+ score**.

**Figure 2:**
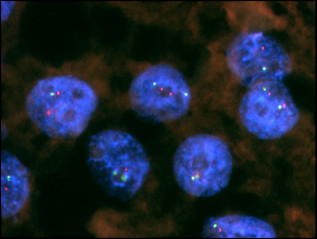
**Fluorescence in situ hybridization analysis of the primary tumor showed a lack of HER-2 amplification, with a ratio of 1.1**.

A 49-year-old premenopausal woman had undergone total mastectomy and sentinel lymph node biopsy for stage I right breast cancer in April 2003. The histological diagnosis was invasive ductal carcinoma of the right breast with no metastasis in one sentinel lymph node. Immunohistochemical (IHC) examinations of the tumor cells showed negative results for both estrogen receptor (ER) and progesterone receptor (PgR) and showed weak membrane staining of HER-2 (1+ score) (Figure 1). Fluorescence in situ hybridization (FISH) analysis found no HER-2 amplification in the primary tumor (ratio, 1.1) (Figure 2). The patient received postoperative adjuvant chemotherapy consisting of 4 cycles of epirubicine 75 mg/m^2 ^and cyclophosphamide 600 mg/m^2 ^every 3 weeks. After the completion of adjuvant chemotherapy, she became postmenopausal and was followed without any treatment.

**Figure 3:**
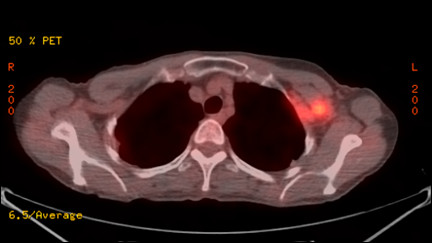
**FDG-PET showed swelling of the left axilla and infraclavicular lymph nodes with FDG accumulation (SUV max 4.5)**. No accumulation was observed in other organs, including the left breast.

**Figure 4:**
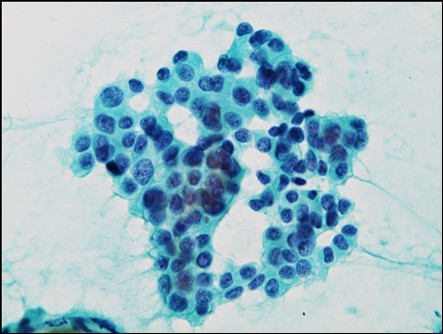
**Fine needle aspiration cytology demonstrated sheet clusters of atypical epithelial cells that showed high a nuclear cytoplasmic ratio, and the appearance of a nucleolus suggests recurrent breast cancer (Papanicolau)**.

**Figure 5:**
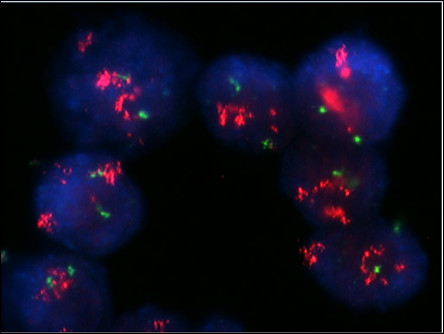
**Fluorescence in situ hybridization (FISH) analysis of the lymph node metastasis showed strong HER-2 amplification in most cells, with a FISH ratio of 5.6**.

Six years and 10 months after primary surgery, she noticed lumps in her left axilla. An ultrasonography and computed tomography (CT) scan confirmed left axillary and infraclavicular lymph node swellings. FDG-PET (2-[18F]Fluoro-2-deoxyglucose positron emission tomography) showed an accumulation of FDG in the left axilla and infraclavicular lymph nodes (Figure 3). The patient subsequently underwent ultrasonography-guided fine needle aspiration (FNA) cytology of the left axilla lymph node (Figure 4). Cytological findings revealed breast cancer metastases and FISH analysis of FNA samples showed *HER-2 *gene amplification (ratio 5.7) (Figure 5). Immunohistochemical examinations of FNA sample showed positive results for both ER and PgR. A PET-CT scan did not reveal any other metastases, and no malignancies were detected in any other organs, including her left breast. With these clinical and cytological findings, she was diagnosed as having HER-2-positive recurrent breast cancer after primary treatment of right triple-negative breast cancer. At this point, there was the possibility of left axillary lymph node metastases from right breast cancer or left occult breast cancer. Initially, we decided to perform chemotherapy before surgery to evaluate the responsiveness to chemotherapy.

**Figure 6:**
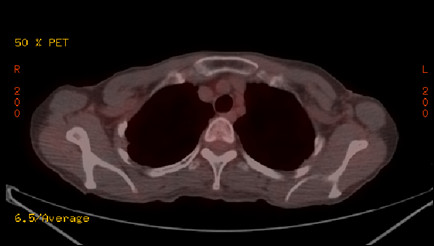
**After administration of three cycles of combination chemotherapy using weekly paclitaxel and trastuzumab, the lymph node swelling disappeared and there was no apparent accumulation of FDG**.

She was treated with weekly paclitaxel (80 mg/m^2^, 3 weeks on, 1 week off) in combination with weekly trastuzumab (initially 4 mg/kg followed by 2 mg/kg every week). After 3 courses of administrations, a PET-CT scan showed complete remission of swelling lymph nodes and no accumulation of FDG was detected (Figure 6). The elevated CEA was normalized. Complete remission of disease was maintained for more than 4 weeks with normalized tumor markers and she was diagnosed as having a complete clinical response by RECIST criteria. No severe adverse event above grade 3 was noted (alopecia grade 1, neutropenia grade 2). She underwent the combination chemotherapy of weekly paclitaxel and trastuzumab for 1 year and continues to showe a complete remission of disease with tolerable neuropathy. Monotherapy with trastuzumab alone chemotherapy is ongoing and additional radiotherapy and surgery are being considered to confirm the local control.

## Discussion

With the introduction of molecular targeting therapies, such as those utilizing monoclonal antibodies or small-molecule tyrosine kinase inhibitors directed to HER2 signaling and hormonal agents, hormone receptor expression and HER-2 status have become the most important prognostic and predictive factors in breast cancer [8-10]. It is currently recommended that these biomarkers be routinely evaluated in every case of primary invasive breast cancer [1,5]. Meanwhile, confirmatory biopsy and reevaluation of biomarkers are not mandatory, and biopsy of metastatic lesions is rarely performed, especially for distant metastases. Thus, the decision to carry out systemic treatment for recurrent and metastatic breast cancer is based on the ER, PgR, and HER-2 status of the primary tumor. However, a substantial proportion of patients with metastatic breast cancer have been reported to have discordances in the expression of these biomarkers between primary and metastatic disease in previous studies. Recent retrospective reviews have shown discordance between the primary tumor and metastases in 14%-48% for ER and PgR status, and in 2%-13% for HER-2 status. A prospective study also demonstrated changes in hormone receptor status in 40% and in HER-2 status in 8% of patients with metastatic breast cancer [1-3,5]. Most importantly, patients with discordant expression of these receptors have been reported to show a decreased rate of post-recurrence survival due to inappropriate use of targeted therapies [2,4]. Considering the therapeutic impact of targeted therapies in breast cancer, misunderstanding in the receptor profile of metastatic lesions can lead to suboptimal therapy and decreased survival.

Compared with hormone receptor status, discordance in the HER-2 status between primary and metastatic lesions is less common, and the impact of this discordance on the prognosis of recurrent breast cancer is less clear [6,7]. However, with the introduction of trastuzumab, the HER-2 status of metastatic disease has become one of the most important predictive and prognostic factors in patients with recurrent breast cancer. Clinical trials have shown a significant survival benefit from trastuzumab in addition to the chemotherapy agent in HER-2-positive advanced breast cancer [11,12]. An institutional-based review also showed that women with HER-2-positive disease who received trastuzumab had an improved prognosis compared with women who did not receive such treatment [13]. However, it is less clear whether the reevaluation of HER -2 status in metastatic lesions has an impact on the prognosis of patients with recurrent breast cancer. In this case report, the patient was initially diagnosed with triple-negative breast cancer, but a reevaluation of the metastatic disease showed her to be HER-2-positive by FISH, and she was diagnosed with HER-2-positive metastatic breast cancer. Based on these findings, she was administered combination chemotherapy using weekly paclitaxel and trastuzumab, and clinically complete remission was immediately achieved and maintained for over 1 year. She is currently disease-free, and an excellent prognosis is expected. The incidence of a discordance in HER-2 status between primary tumor and recurrent disease may be small, but an altered HER-2 status could have substantial clinical impact in patients with recurrent breast cancer with the introduction of HER-2-targeting agents. With these findings, sampling and assessment of the metastatic lesion should be considered in patients with recurrent disease without excessive involvement.

There are some limitations to this case report. First, one cannot completely deny the possibility of left occult breast cancer with axillary lymph node metastases. Although the frequency of occult breast cancer is reportedly small, accounting for less than 1% of all breast cancers [14], and no lesion was detected in her left breast by detailed examination at the diagnosis of recurrent breast cancer, there remains a small possibility of occult HER-2-positive left breast cancer with regional lymph node metastases. Second, in this case, *HER-2 *gene amplification of recurrent disease was detected in samples of fine needle aspirates by HER-2 FISH. In a daily setting, HER-2 FISH is performed on tissue sections according to published guidelines [15]. There could be a discrepancy in *HER2 *gene amplification between tissue and cytological samples. However, it has previously been reported that *HER2 *amplification in FNA samples shows a good correlation with the findings of FISH and IHC results from corresponding histological sections [16]. In our institute, we have also confirmed the concordance between tissues and cytology samples in HER-2 FISH results. Thus, the HER-2 FISH results from FNA samples in this case correctly revealed the HER-2 status of metastatic disease.

## Conclusion

We observed a case of HER-2-positive recurrent breast cancer showing a clinically complete response to trastuzumab-containing chemotherapy after primary treatment of triple-negative breast cancer. Reassessment of the HER-2 status of metastatic lesions should be considered in all patients without excessive involvement.

## Consent

Written informed consent was obtained from the patient for publication of this Case report and any accompanying images. A copy of the written consent is available for review by the Editor-in-Chief of this journal.

## Abbreviations

immunohistochemistry: IHC; fluorescence in situ hybridization: FISH; estrogen receptor: ER; progesterone receptor: PgR; computed tomography: CT; 2-[18F]Fluoro-2-deoxyglucose positron emission tomography: FDG-PET; fine needle aspiration: FNA.

## Competing interests

The authors declare that they have no competing interests.

## Authors' contributions

HS, YK, TS and NM tracked the clinical data and drafted the manuscript. TK, KK, TS, AE, RH and TK participated in the design of the study and revised the manuscript. Pathologists MO and KA evaluated the results of the immunohistochemical analysis and fluorescence in situ hybridization. MO conceived of the study, participated in its design and coordination, and revised the manuscript for important intellectual content. All authors read and approved the final manuscript.
